# Cell autocloning as a pathway to their real rejuvenation

**DOI:** 10.3389/fragi.2024.1429156

**Published:** 2024-07-29

**Authors:** Lev Salnikov

**Affiliations:** AntiCA Biomed, San Diego, CA, United States

**Keywords:** aging, geroprotection, rejuvenation, polyploidy, asymmetric cell division

## Abstract

The article gives a brief description of geroprotection and rejuvenation methods known to date, presenting their main mechanisms and limitations. To overcome the main limitations of the process of rejuvenation, it is possible to use a process called “cell autocloning.” The principle of the proposed method of rejuvenation is as follows: a periodic process of autocloning of the cell nucleus is initiated in the cellular genome with the formation of one unstable daughter copy and its subsequent self-elimination. In this case, the process of cell division stops in the phase of nuclei divergence without subsequent physical separation of the cell itself. This is especially important for postmitotic cells, where the looping of the “unidirectional” line of the ontogenesis program into a “ring” will mean their transition into renewable cells. The prototype for autocloning mechanisms could be the already known ways in which cells adapt to the increasing amount of their damage over time. These are polyploidy and asymmetric cell division, relying on which it is possible to obtain a renewable process of cell nuclei division, when only the original nucleus remains as a result of division. Although this is not a simple task, there are possible pathways to its solution using approaches that can suggest modern knowledge from the field of molecular and cell biology and genetics. The realization of such a goal will require a lot of work, but the expected result justifies it.

## Introduction

The very concept of rejuvenation means an impact aimed essentially at trying to transform an old organism into a young one (Zhang et al., 2022). The success of such effects is directly related to our level of understanding of aging mechanisms. Here it is necessary to emphasize the difference between rejuvenation and geroprotection. If in the first case the goal is radical rejuvenation of the whole organism, the essence of geroprotection is to slow down involutional processes as much as possible, to reduce the rate of progression of this or that pathology, to delay the time of occurrence of unfavorable changes. Geroprotection is aimed not at the root cause of aging, but at individual signs and mechanisms associated with aging. In addition, the effect of geroprotective effects is transient (Salnikov and Baramiya, 2021). Success in the pathway of rejuvenation requires an understanding of the causes and basic mechanisms of aging. The problem of understanding the processes of aging remains important, despite the huge number of works devoted to this topic (de Magalhães, 2024). From our point of view, it is necessary to distinguish between the causes and mechanisms of realization of this process. Aging itself manifests itself at the level of multicellular organisms, hence its main mechanisms are realized exactly where a cell receives in addition to its needs the necessity to perform organismal functions (Salnikov and Baramiya, 2020). When considering the causes of aging, we assume that the main cause of aging is embedded in the very “design” or principle of multicellular organization. Let us briefly explain this statement. Evolutionarily, multicellular organisms developed on the basis of unicellular colonies, gradually acquiring new genes that provide integrative functions peculiar to the organism. At the same time, the cells that make up the organism retain their original genes that provide all their basic needs. Thus, in the cells of an organism it is always possible to distinguish an evolutionarily conservative part of the genome responsible for providing cellular infrastructure, i.e., housekeeping genes (HG), and an evolutionarily active part of the genome providing all organismal functions, i.e., integrative genes (IntG). The described ratios of the functional parts of the cellular genome, were shown by us when analyzing the age-dependent production of their RNA (Salnikov et al., 2023; Salnikov et al., 2024). We emphasize that only the IntG part of the genome is regulated by the developmental program, or ontogenesis (Salnikov, 2022). Thus, the cells of the organism are constantly facing a choice: either slowing down the rate of division, leading to a decrease in the cost of their own repair, or transition to uncontrolled division, which is essentially carcinogenesis.

Proponents of the entropic theory of aging emphasize (Perevoshchikova and Fedichev, 2024) that entropy increases with age both in the organism as a whole and in its individual cells, without commenting on the practical “immortality” of single-cell or tumor cells. It turns out that the very aggregation of cells into an organism causes aging processes. Also, like aging, carcinogenesis is the price to pay for multicellularity. Oncogenesis is of great interest for understanding of aging processes due to one important fact - cells that give rise to cancer are immortal. The HeLa line has been maintained for seven decades without showing signs of degradation. In fact, we observe it as a single-cell culture (Salnikov and Baramiya, 2021). The genome of differentiated cells of an organism is “designed” by evolution in such a way that in prophase, or after mitosis is completed, the activity of the genome is switched to fulfill organismal functions, creating over time a deficit of resources needed by the cell for repair. Maintaining the repair balance depends on the rate of division for each tissue, or the ratio of stimulatory and inhibitory signals in the cell - switching from HG to IntG at the tissue and cellular levels. Each tissue type has its own program for achieving the necessary maturity. Their total work creates the aging trajectory (Gems et al., 2023), in which highly differentiated cells play an important role. These are the cells from which senescent cells are formed in the course of aging, which are also a target for geroprotective interventions. It is important to note that the very definition of senescent cells (Moiseeva et al., 2023) always refers to postmitotic, or rarely dividing cells with low mitotic index (MI). The organism tissues consisting of such highly differentiated cells play not only a major role in the functioning of the organism, but also in aging processes. The increasing number of senescent cells with a deficit of reparative capabilities that occurs with age naturally leads to the accumulation of cytotoxic products and associated metabolic disorders in them and in their environment (Nelson and Masel, 2017). As a result, tissues with high MI are subjected to increasing metabolic pressure from an increasing mass of slowly dividing and postmitotic cells. For actively dividing cells of constantly renewing tissues like bone marrow, intestinal and mucous membrane epithelium, and the growth layer of the skin, such toxic exposure leads to a gradual decrease in their stem cell numbers and a reduced rate of regeneration. Agreeing with the definition of the phenomenon “aging” itself. As a phenomenon of incomplete recovery or repair of cellular disorders arising during life (Lagunas-Rangel and Bermúdez-Cruz, 2019; Ferreira et al., 2021; Salnikov 2022), we obtain a reliable criterion for assessing any effects aimed at rejuvenation.

### Cells autocloning

The main conclusion from our ideas about the causes of aging is that there is no direct connection between the function of the infrastructural part of the genome and specialized genes controlled by the ontogenesis program (Salnikov and Baramiya, 2020). In nature, we can observe ways in which cells adapt to the increasing amount of their damage over time. In multicellular organisms, we observe the phenomenon of polyploidy or multinucleation in highly differentiated cells, most commonly observed in hepatocytes (Donne et al., 2020; Bailey et al., 2021; Fang et al., 2023). Although polyploidy is designed to compensate for the decrease in repair, at the same time cell size increases. The size increase itself largely negates the result obtained due to polyploidy and is one of the signs of aging (Lanz et al., 2022). There is another mechanism of cell adaptation to the accumulation of metabolically inert or harmful products in cells. In unicellular organisms, in addition to natural selection that eliminates unsuccessful cell strains, we can note the process of asymmetric division (Chhabra and Booth, 2021), in which one of the daughter cells concentrates the main amount of damaged intracellular substance into one of its daughter cells, followed by the death of this cell. Such a mechanism of elimination of unfavorable changes in the cells of the organism is available only during regeneration, where this method loses its significance, although it is of great interest from our point of view.

**FIGURE 1:**
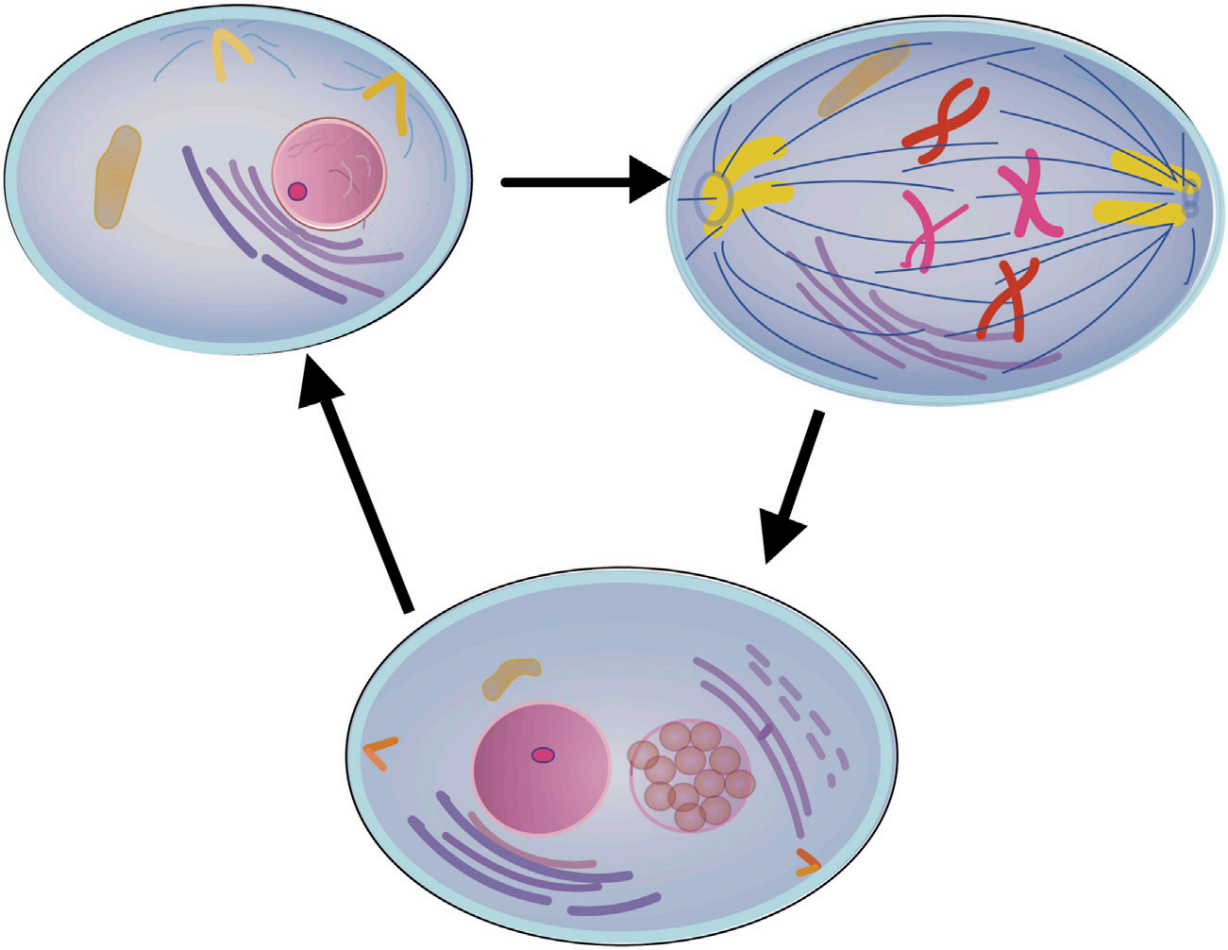
Schematic of the cell autocloning process.

Recall that any cell is adapted by evolution to self-preservation through the process of division. Decrease in the frequency of division and its stoppage over time always leads to cell death. Based on the known ways of maintaining cellular repair, let us present a way to achieve cellular self-renewal. It is possible to overcome the main limitations of the process of rejuvenation related to the specific features of highly differentiated cells of the organism by means of the process called “cell autocloning”. The principle of the proposed method of rejuvenation is as follows: a periodic process of autocloning of the cell nucleus is initiated in the cell genome with the formation of one unstable daughter copy and its subsequent self-liquidation. In this case, the process of cell division stops in the phase of nuclei divergence without subsequent physical separation of the cell itself. This is especially important for postmitotic cells, where the looping of the “unidirectional” line of the ontogenesis program into a “ring” will mean their transition into renewable cells. This makes it possible not only to periodically intensify repair processes in the cell nucleus itself when restarting the differentiation process, but also to restart active self-renewal in the cell cytoplasm without disturbing its spatial structure, which is especially important for postmitotic cells, especially such as neurons. The essence of the proposed method is represented schematically in Figure 1.

The cellular genome initiates a periodic process of autocloning of the cell nucleus with the formation of an unstable daughter copy and its subsequent self-liquidation without physical separation of the cell itself.

To start the autocloning process, it is necessary to build into the cellular genome a mechanism for its periodic initiation, responding to the signal of a certain molecular trigger, which can be external, periodically introduced into the organism or internal, simply produced by it. A necessary result of cell nuclear division necessary for autocloning, when only the original nucleus remains as a result of division, may be the production of DNA polymerase incapable of full synthesis, leaving ultimately only the original nucleus DNA. The synthesis of such defective DNA polymerase must be tightly bound and follows the activation of autocloning itself, stopping the process of mitosis in prophase.

The internal trigger for the autocloning process may be a certain concentration of a cellular product, possibly related to the organismal function of the cell itself. A difference in the concentration of a certain intracellular product, within different parts of the cell itself, also can be used. This difference may serve as a trigger for autolysis of one of the daughter nuclei, which has fallen into the zone of its increased concentration. It is important to note that the proposed mechanism for triggering autocloning should function separately from the natural, existing in the cells of the organism their division process. This will not interfere in the process of cell maturation and metabolism in rapidly dividing tissues, as well as in the course of tissue regeneration.

Although this is not a simple task, there are possible pathways to its solution using approaches that can suggest modern knowledge from the field of molecular and cell biology. In particular, the data obtained from polyploidy studies should help to establish a mechanism for periodic division of the nucleus without the physical process of cell separation. In-depth study of asymmetric transfer and activation of traits occurring in the process of development and formation of multicellular organisms may form the basis for the mechanism of self-destruction of one of the daughter nuclei in a cell, and the successes achieved in genome editing will allow include the genetic information necessary for the proposed method.

## Discussion

A number of geroprotective methods are currently known to have some results in increasing longevity and improving the aging organism (Melo Pereira et al., 2019). One of the first geroprotective methods discovered by researchers can be considered the calorie restriction method (Lan et al., 2019; Ealey et al., 2024; Wang et al., 2024). Using this method for the first time on the model organism of mice it was possible to obtain a reliable increase in their life expectancy by slowing down their growth and maturation, although on humans it was not possible to achieve a similar result (Wei et al., 2024). Currently, in this direction, studies of drugs aimed at limiting the intensity of metabolism, including the use of rapamycin (Pelton, 2022), which have a geroprotective effect (Sabini et al., 2023), are underway. It should be noted that attempts to slow down cellular metabolism, even if successful, simply slow down the ontogenesis program and can only delay the aging processes without affecting their underlying cause. The geroprotection effect is also aimed at the development of drugs aimed at stimulating the work of lysosomes to eliminate the accumulation of metabolically inert or harmful products accumulated in the cell with age (Sun et al., 2020). The works devoted to the study and prospects of the method of “self-purification” of the cell using microvesicles are close to this direction (Pattabiraman and Kaganovich, 2014). In these approaches, intensification of the processes of cellular products decay or acceleration of their release is also a temporary measure that cannot stop the decrease in reparative capabilities of cells. Another method of geroprotection is aimed at using cellular signals isolated from the blood plasma of young organisms. The method is designed to compensate for the deficiency of cellular signals for growth and repair that arise in old cells (Mitteldorf, 2023; Chen et al., 2024; Fennel et al., 2024). Such an approach to geroprotection will have prospects only in case of isolation of specific cell signals stimulating cell repair, but also, as well as the previous methods, is not able to affect the implementation of the ontogenesis program in cells. Separately it is necessary to note the direction in geroprotection connected with attempts to influence or eliminate senile cells (Tang et al., 2024; Wan et al., 2024), which not only play an important role in aging processes, but simply mechanically do not allow to replace themselves with new healthy cells. A disadvantage of this method is the inability of highly differentiated cells to divide and replace old cells, although it is possible to improve the results of this method when combined with cellular reprogramming (Mahmoudi et al., 2019; Chen and Skutella, 2022). The most promising and attracting the most attention of researchers is the method of cellular reprogramming, which uses epigenetic manipulations to return cells to the state of pluripotency (Chiavellini et al., 2021; Lu et al., 2023). In essence, the method is designed to temporarily reverse cell differentiation. Some successes have been achieved on this pathway, but mainly at the level of individual tissues of the organism (Matteini et al., 2024; Mitchell et al., 2024; Pozzo et al., 2024). Although cellular reprogramming is at the beginning of the pathway to its use at the level of the whole organism, this approach is initially aimed at real rejuvenation (Simpson et al., 2021).

Summarizing the presented data on methods of geroprotection we can conclude that practically all methods aimed at life extension are reduced to attempts to keep the organism in the phase of development by prolonging the period of its growth, be it calorie restriction, enhanced removal of cellular debris, exposure to growth factors Cellular reprogramming is the only method aimed at stopping unidirectional ontogenesis, allowing to return the cells to the necessary level of their own repair. It should be noted that the main disadvantages of the currently known methods of rejuvenation are not only the necessity of constant external influence, but also the inability to effectively replace old functionally inefficient cells. For postmitotic cells, whose rejuvenation is impossible by regeneration, it is necessary to solve the problem of ''division without division”, or how to make them rebuild themselves (Baramiya et al., 2020). In the following, we will present one of the possible pathways to achieve such a result.

The cell adaptation mechanisms we have already referred to, allow us to briefly present pathways that could be used to realize the autocloning process in the future. Although a detailed discussion of the ways to create and operate such autocloning mechanisms is not the purpose of this work, some assumptions can already be made. In the search for mechanisms that may become the basis for autocloning, attention should be paid to the features of asymmetric cell division, which exhibits not only a shifted segregation of macromolecules (Sunchu and Cabernard, 2020), but also an asymmetry of histone distribution between the nuclei of daughter cells (Xie et al., 2017). Nuclear polarization of transcription factors during mitosis has been shown to set parental and daughter cell fates (Rujano et al., 2006; Liu et al., 2018). In addition, the ability to distinguish “old” from “new” and asymmetrically segregate DNA is inherent even in simple unicellular eukaryotes (Anda et al., 2021). Attention should also be paid to mitotic recombination, or the exchange of genetic material between sisters chromatids during mitosis. If the altered gene is present on one of the sister chromatids, recombination can result in asymmetric transfer of the altered gene to only one of the daughter nuclei. Thus, histones or cellular mechanisms may affect the segregation of specific chromosomes or chromosomal regions during mitosis. These mechanisms may favor the segregation of the altered gene into one of the daughter nuclei. Building on the results of studies focusing on different factors regulating cell division (Salazar-Roa and Malumbres, 2017; Üretmen Kagıalı et al., 2017; Ong and Torres, 2019; Matthews et al., 2022) as well as the previously presented work on polyploidy, also holds promise in developing the mechanisms required to trigger the cellular autocloning process. Note that there is a possibility of using the concentration level of different cellular products as triggers for their autocloning process, as well as the possibility of an asymmetric response to such a trigger due to concentration differences in different parts of the cell.

In conclusion, we emphasize that the method of rejuvenation described in this paper is currently only a strategic direction, which is quite achievable for the current level of biotechnology development. In case of its creation, true rejuvenation allows not only to significantly increase life expectancy, but also to avoid age-related diseases due to normalization of reparative activity in the cells of the organism, returning the organism to the optimal level of its adaptive capabilities. At the same time, the presented direction will allow not only to avoid the disadvantages inherent in the existing methods of geroprotection, but also to replace them. It will take a lot of work to realize such a goal, but the intended result is totally worth it.

## Data Availability

The original contributions presented in the study are included in the article/Supplementary material, further inquiries can be directed to the corresponding author.
